# Dual-Band Planar Microwave Solid Complex Dielectric Constant Sensor System Based on E-Interdigital Structure

**DOI:** 10.3390/s25185789

**Published:** 2025-09-17

**Authors:** Haoyang Shi, Xuchun Zhang, Lin Huang, Kun Wang, Zanyang Wang

**Affiliations:** Air Defense and Anti-Missile Academy, Air Force Engineering University, Xi’an 710043, China; shy2227128394@163.com (H.S.); linhuang125@163.com (L.H.); 17765073967@163.com (K.W.); wzy_098@163.com (Z.W.)

**Keywords:** microwave sensor, complex dielectric constant, dual-band, sensor system

## Abstract

This paper introduces a dual-band planar microwave sensor system for measuring the complex dielectric constant of solid material. The sensor system comprises three constituent parts: the sensing probe, the circuit module and the broadband coupler. The sensing probe is composed of a host part and a sensing area. The host part is composed of a microstrip line, which facilitates system integration with other planar microwave components. The sensing area comprises two pairs of E-interdigital structures, which were originally developed from the interdigital capacitor. This configuration manifests two resonant frequency points, specifically 3 GHz and 3.92 GHz. Consequently, any environmental effects exhibit equivalent variation at both resonant frequency points, thereby substantiating the efficacy of the proposed sensor system for differential operation, which has the capacity to mitigate the impact of environmental conditions. The circuit module comprises a controller, two detectors and a signal generator, which facilitate the generation and processing of radio frequency signals within the system. The function of the broadband coupler is to differentiate between the incident signal and the reflected signal. The operating principle is predicated on the variation in the resonant frequency and peak attenuation with respect to the complex dielectric constant of the material under test (MUT). In order to validate the effectiveness of the proposed sensor system, a prototype is fabricated and tested. The proposed sensor system is distinguished by its high sensitivity and low cost. The apparatus is capable of performing measurements independently and without the necessity for auxiliary equipment.

## 1. Introduction

The characterization of insulating materials is of paramount importance in industrial applications, particularly in the field of material science [1]. Despite the existence of numerous criteria for material characterization, the most significant parameter is the complex dielectric constant $\left(\varepsilon_{r}=\varepsilon_{r}^{\prime }-j\varepsilon_{r}^{\prime \prime }\right)$. The real part is typically referred to as the dielectric constant, while the imaginary part is known as the loss factor. This factor is indicative of the material’s behavior and is closely related to other physical and chemical properties. Consequently, it can facilitate an accurate characterization of the material [2]. Common techniques for measuring the complex dielectric constant of materials include microelectrochemical systems (MEMSs) [3], optical measurement [4], and infrared measurement [5]. Optical and infrared measurement techniques are commonly utilized to determine the refractive index and absorption coefficient of materials. While these techniques can also assess the complex dielectric constant of materials, their practical application is limited due to factors such as high costs, intricate principles, and invasive nature. The measurement of the complex dielectric constant by microwave has attracted considerable attention from researchers in recent years [6,7,8,9]. The microwave measurement method is experiencing a surge in popularity, owing to its numerous advantages, including its compact size, high sensitivity, reusability, non-destructive detection, and a range of other features. The microwave measurement method encompasses a range of approaches, including the coaxial probe method, the free space method, the parallel plate method, and the planar microwave resonance method, among others [10]. The planar microwave resonance method has been demonstrated to be a viable solution to the system compatibility problem. The method is based on the change in resonant frequency and peak attenuation, which are caused by the interaction of the resonant structure with the MUT during the measurement process [11,12]. The microwave resonance method has been employed to measure solid thickness [13,14], solid/liquid complex dielectric constant [15,16,17,18,19] and rotation angle [20,21,22]. Currently, there are many structures and technologies for the design and fabrication of planar microwave structures based on the resonance method, such as substrate integrated waveguide (SIW) technology in [23,24,25,26,27] and metamaterial technology for the measurement of complex dielectric constant [28,29], which provide an ideal plane for realizing compact and small microwave sensors with high accuracy, quality factor and sensitivity [30,31,32]. However, it should be noted that the majority of microwave structures in the literature are designed to measure only the real part of the complex dielectric constant, while the imaginary part is generally ignored. Furthermore, auxiliary equipment such as vector network analyzer is utilized to facilitate the measurement process.

This paper introduces a simple and novel prototype system for measuring the complex dielectric constant of solid material. The operating principle of the sensor system is based on the variation in the resonant frequency and peak attenuation with respect to the complex dielectric constant of the solid material. The proposed sensor system is distinguished by its high accuracy and its capacity for independent measurement of the complex dielectric constant of solid materials, thus obviating the need for auxiliary equipment and resulting in a substantial reduction in system cost.

## 2. Proposed Sensing Probe Configuration

### 2.1. The Structure of the Sensing Probe

**Figure 1:**
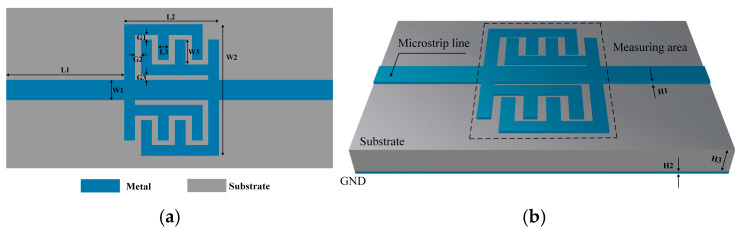
(**a**) The structure of the sensing probe. (**b**) Three-dimensional view of the sensing probe.

The configuration of the sensing probe is illustrated in Figure 1a. Its detailed dimensions are summarized in Table 1 below. The host part is a 50 Ω microstrip line, which is also used to connect the input/output ports, and this well-known simple structure facilitates connection to other planar microwave components. The probe’s sensing area is formed by two pairs of E-interdigital structures, which originally evolve from the interdigital capacitor. These structures are symmetrically arranged on either side of the microstrip line. This configuration has the potential to further enhance the confinement of the electric field. As illustrated in Figure 1b, the sensing probe is depicted in three dimensions. The metal is made of copper with a thickness of 0.018 mm. The substrate is made of F4BM220 with a thickness of 0.8 mm, where the $\varepsilon_{r}^{\prime }=2.2$ and the tanδ=0.001. The relationship between tanδ and $\varepsilon_{r}$ is as follows:

(1) $$\tan\delta =\frac{\varepsilon_{r}^{\prime \prime }}{\varepsilon_{r}^{\prime }}$$

**Figure 2:**
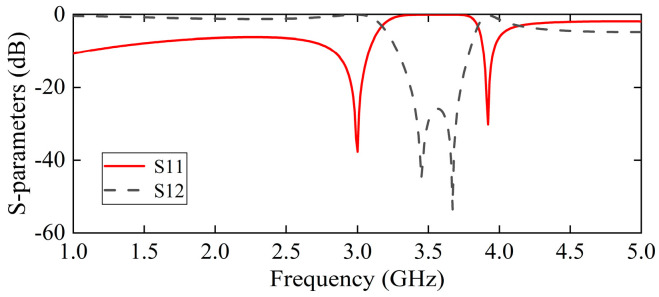
The electromagnetic field simulation results of the scattering parameters of the sensing probe.

**Figure 3:**
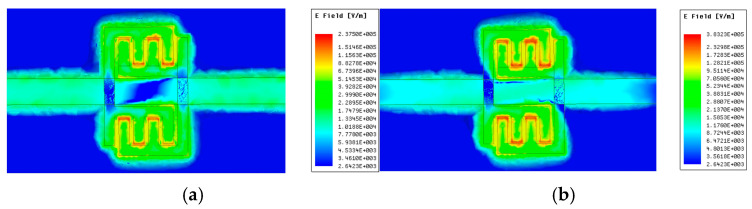
Electric field distribution at (**a**) 3 GHz, and (**b**) 3.92 GHz of the sensing probe at unloaded.

The electromagnetic simulation of the sensing probe was conducted utilizing HFSS 19.0 software. As shown in Figure 2, the reflection coefficient $S_{11}$ has two resonant frequency points, specifically, 3 GHz and 3.92 GHz. The generation of two resonant frequency points within the sensing area occurs simultaneously. Any environmental influence will induce equivalent changes at these two resonant frequency points, effectively mitigating the impact of environmental conditions. For instance, when the environmental temperature varies, the two resonant frequency points may shift. However, through differential operations, the adverse effects caused by this frequency shift can be effectively eliminated. As illustrated in Figure 3a,b, the distribution of the electric field for the sensing probe is demonstrated at two resonant frequency points, respectively. Figure 3 illustrates the electric field distribution on the sensing probe’s top metal structure. At 3 GHz, the electric field density is stronger at the fingertips of the E-interdigital structure; so, the capacitance effect seems to be more pronounced in this section. This finding suggests that the capacitance effect is more significant in this section, leading to the predominance of the effect in this region. At 3.92 GHz, the electric field at the fingertips is more concentrated than at 3 GHz, whilst there is also a significant enhancement in the electric field between the microstrip line and the E-interdigital structure. It is evident that an increase in the strength of the electric field confinement results in a corresponding enhancement in the sensitivity to the material.

**Table 1:** The dimensions of the sensing probe.

Dimensions	Value (mm)	Dimensions	Value (mm)
L1	21.00	G1	0.20
L2	8.00	G2	0.40
L3	1.00	G3	0.20
W1	2.47	H1	0.018
W2	12.27	H2	0.018
W3	2.50	H3	0.80

### 2.2. The Operating Principle and Equivalent Circuit

The primary mode of propagation in a microstrip line is the quasi-TEM mode, which is caused by the inhomogeneity of the dielectric region. Some of the electric field lines exist within the dielectric region between the strip and the ground plane, whereas others extend into the air above the substrate. When the solid material intended for measurement is placed on the sensing area, it interacts with the fringe fields, potentially altering the electrical performance of the structure. In the context of the system composed of the solid material and the probe, a change in the complex dielectric constant of the solid material will lead to a corresponding alteration in the effective complex dielectric constant of the system [33,34]. Consequently, the resonant frequency and peak attenuation of the sensor will be affected by variations in the complex dielectric constant of the solid material. The complex dielectric constant of the material is determined by performing parameter inversion based on the detection of the resonant frequency and peak attenuation of the sensor.

**Figure 4:**
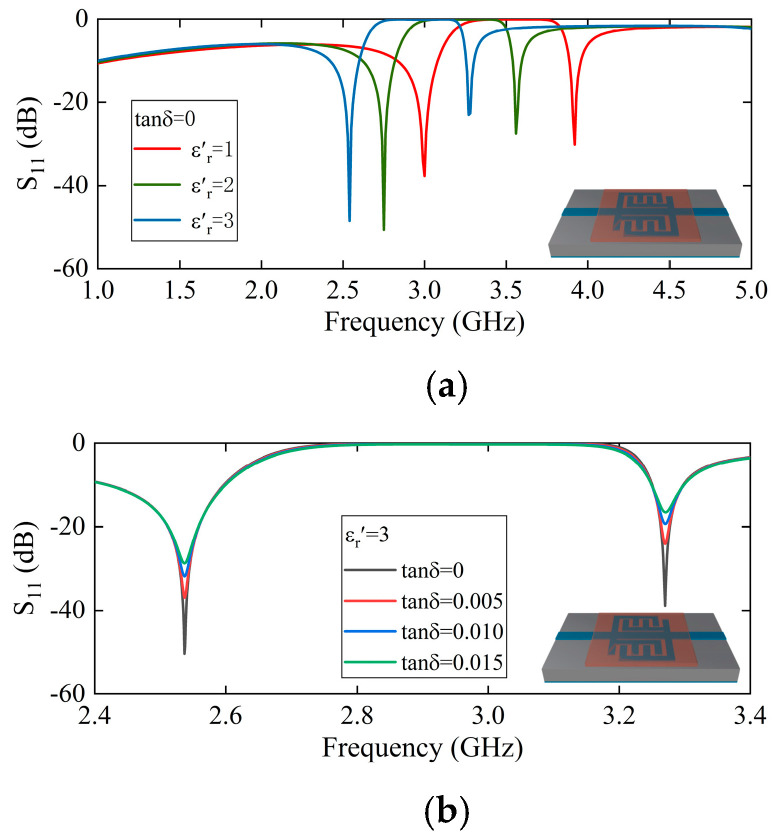
(**a**) $S_{11}$ varies with the $\varepsilon_{r}^{\prime }$ when tanδ=0. (**b**) $S_{11}$ varies with the tanδ when $\varepsilon_{r}^{\prime }=3$.

As illustrated in Figure 4, the simulation outcome demonstrates the reflection coefficient $S_{11}$ of the sensing probe when subjected to a range of solid materials. The size of the solid sample is designated as 15 mm×15 mm×0.5 mm, ensuring that its surface area exceeds that of the sensing area of the probe. This design guarantees full coverage of the sensing area. Furthermore, the center of the solid material aligns with the center of the sensing area, and it is uniformly affixed to the sensing probe from both the left and right sides. As long as the solid material can cover the sensing area, variations in the area of the solid material will not impact the resonant frequency point. However, changes in the solid material thickness will alter the electromagnetic field distribution within the sensing area, necessitating a re-fitting process. In Figure 4a, when the tanδ=0, both resonant frequency points decrease as the $\varepsilon_{r}^{\prime }$ increases. In Figure 4b, when the $\varepsilon_{r}^{\prime }=3$, the two resonant frequency points do not shift but the peak attenuation decreases as the tanδ increases. Therefore, the complex dielectric constant of the material can be extracted from the changes in the resonant frequency and the peak attenuation of the sensing probe.

**Figure 5:**
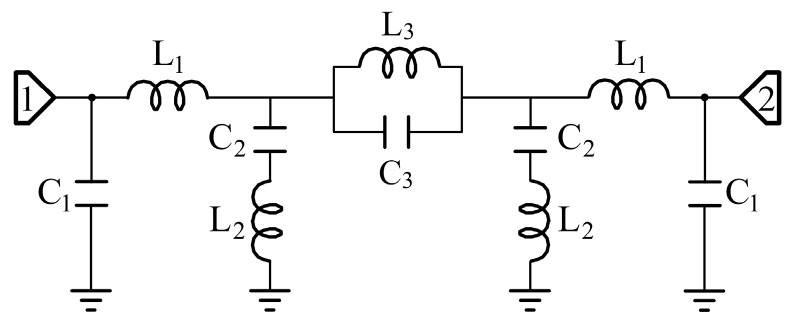
Equivalent circuit model of the sensing probe.

**Figure 6:**
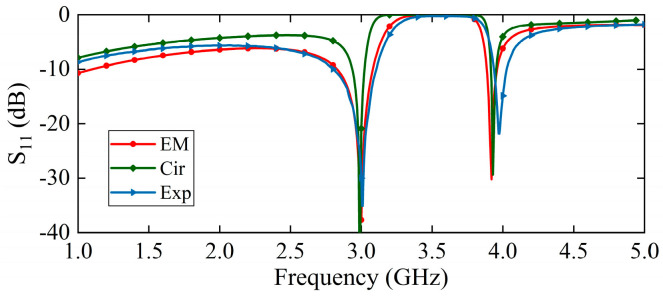
Comparison of electromagnetic field simulation, circuit simulation and experimental testing under no-load conditions.

The equivalent circuit model of the sensing probe is shown in Figure 5. The input and output of the microstrip lines are simulated by these two sets of inductors $L_{1}$ and capacitors $C_{1}$. The resonant circuit formed by two pairs of E-interdigital structures and the ground is represented by two sets of series inductors $L_{2}$ and capacitors $C_{2}$. A parallel resonant loop consisting of inductor $L_{3}$ and capacitor $C_{3}$ is used to simulate the mutual coupling effect between the E-interdigital structures. The parameters of the equivalent circuit model are $L_{1}=1.29\;\mathrm{nH}$, $C_{1}=0.97\;\mathrm{pF}$, $L_{2}=1.80\;\mathrm{nH}$, $C_{2}=1.05\;\mathrm{pF}$, $L_{3}=0.19\;\mathrm{nH}$, $C_{3}=12.51\;\mathrm{pF}$. Placing the solid material to be measured above the sensing area changes the effective complex dielectric constant of the system, which is reflected in the equivalent circuit by changing the values of the parameters of the series resonance loop. The greater the complex dielectric constant of the solid material, the greater the value of $L_{3}$ and $C_{3}$. As illustrated in Figure 6, a comparison is made between electromagnetic field simulation, circuit simulation and experimental testing under no-load conditions. It is evident that the three methods demonstrate a satisfactory degree of consistency.

## 3. Measurement Details of the Dielectric Constant

As demonstrated by the above principle of operation of the sensing probe, a change in the loss tangent alone will not have any effect on the resonant frequency point, but only on the peak attenuation. The observed change in the dielectric constant is consistent with the shift in the resonant frequency point. Consequently, the dielectric constant and the loss tangent can be extracted using the shift in the resonant frequency point and the change in peak attenuation, respectively.

### 3.1. The Polynomial Fitting of the $\varepsilon_{r}^{\prime }$

**Figure 7:**
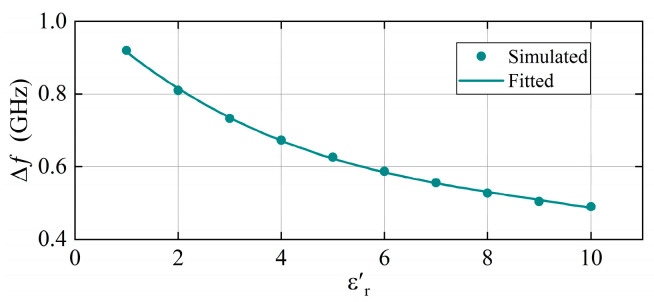
The polynomial fitting of the frequency difference.

As shown in Figure 7, since tanδ does not change the resonant frequency point, the effect of $\varepsilon_{r}^{\prime }$ on the resonant frequency point is simulated assuming tanδ=0. During the simulation process, the dielectric constant of the materials was varied from 1 to 10. This range encompasses the dielectric constants of most common insulating materials. Relative to the simulation results, a polynomial curve-fitting model is used to derive an explicit formula correlating $\varepsilon_{r}^{\prime }$ with the resonant frequency point. The Δf represents the frequency difference between the high-frequency resonant point and the low-frequency resonant point. Using the frequency difference to characterize the dielectric constant can effectively eliminate the influence of environmental factors. The dot traces in the figure represent the simulation results and the curves represent the polynomial fitting results. The fitting equation is as follows:

(2) $$\Delta f=-4\times 10^{-4}{\left(\varepsilon_{r}^{\prime }\right)}^{3}+1.28\times 10^{-2}{\left(\varepsilon_{r}^{\prime }\right)}^{2}-0.135\varepsilon_{r}^{\prime }+1.04$$

where the fit correlation $R^{2}$ of the curve reached 0.9993. Figure 7 shows that Δf decreases with an increase in the dielectric constant. This indicates that the resolution will decrease when testing materials with high dielectric constants. However, within the testing range where $\varepsilon_{r}^{\prime }<10$, Δf can still maintain a value above 0.5 GHz, suggesting that the overall resolution remains relatively high.

### 3.2. The Rational Taylor Fitting of the tanδ

In comparison with low-frequency resonant points, high-frequency resonant points exhibit superior electric field concentration capabilities and heightened sensitivity to alterations in material parameters. Consequently, the high-frequency resonant points are selected for the calculation of loss tangent. It can be seen in the simulation results of Figure 4b that when the $\varepsilon_{r}^{\prime }$ is constant, the variation in the tanδ affects the peak attenuation. The variation in the peak attenuation affects the quality factor $Q_{\mathrm{MUT}}$ of the measurement system, which is given by the following equation:

(3) $$Q_{\mathrm{MUT}}=Q_{U}\left[1-10^{\frac{S_{11}\left(\mathrm{dB}\right)}{20}}\right]$$

where $Q_{U}$ is the unloaded quality factor, which is calculated from the ratio of the unloaded resonant frequency point to the −3 dB bandwidth.

**Figure 8:**
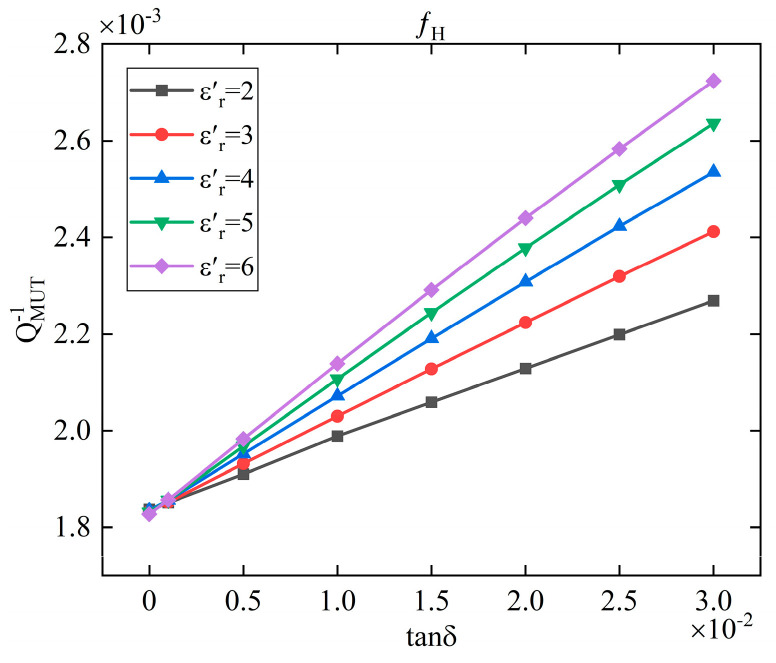
The relationship between $Q_{\mathrm{MUT}}^{-1}$ and tanδ at the high-frequency resonant point.

**Figure 9:**
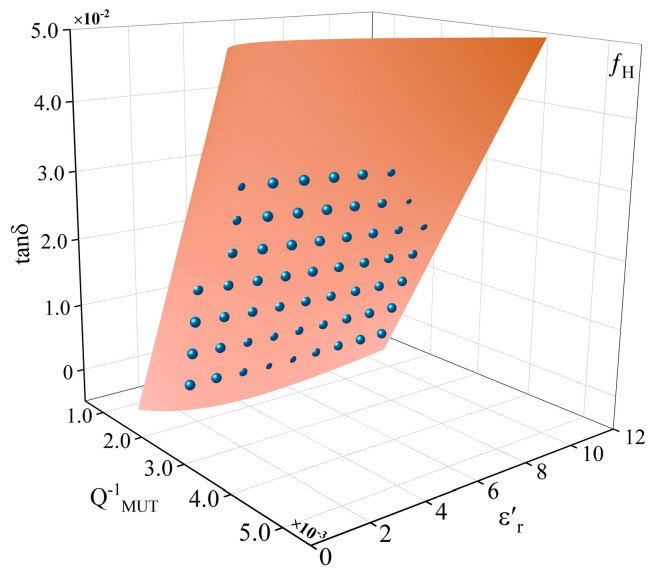
The Rational Taylor fitting of the tanδ at the high-frequency resonant point.

Figure 8 shows the relationship between $Q_{\mathrm{MUT}}^{-1}$ and tanδ at different $\varepsilon_{r}^{\prime }$. It can be seen that the relationship between $Q_{\mathrm{MUT}}^{-1}$ and tanδ is linear, but the linear ratio is different when the $\varepsilon_{r}^{\prime }$ is different. In order to fit the three interrelated parameters together, in this paper, a three-dimensional rectangular coordinate system is set up and the Rational Taylor model is selected to fit the parameters, as shown in Figure 9. The model uses a combination of rational Bezier curves and Taylor series to represent the surface, which has the advantage of being more flexible in representing the local geometry of the surface, allowing for a better approximation of very complex geometries. The ability to fine-tune the local geometry at any point on the surface through the Taylor series makes the Rational Taylor surface model ideal for applications requiring high precision control. As the model requires the computation of Taylor series, this is more demanding on computational resources. However, for the fitting scenarios in this paper, the effect of computational complexity is negligible. The loss tangent can be calculated by:

(4) $$\tan\delta =\frac{F\left(Q_{\mathrm{MUT}}^{-1},\varepsilon_{r}^{\prime }\right)}{G\left(Q_{\mathrm{MUT}}^{-1},\varepsilon_{r}^{\prime }\right)}$$

For the high-frequency resonant point, the following equations are used:

(5) $$\begin{array}{ll} F\left(Q_{\mathrm{MUT}}^{-1},\varepsilon_{r}^{\prime }\right)= & -0.006-0.02\varepsilon_{r}^{\prime }+11.37Q_{\mathrm{MUT}}^{-1}\\ & +8.78\varepsilon_{r}^{\prime }\cdot Q_{\mathrm{MUT}}^{-1}+43.61Q_{\mathrm{MUT}}^{-2} \end{array}$$

(6) $$\begin{array}{ll} G\left(Q_{\mathrm{MUT}}^{-1},\varepsilon_{r}^{\prime }\right)= & 1-0.25\varepsilon_{r}^{\prime }+1.96Q_{\mathrm{MUT}}^{-1}+0.06{\left(\varepsilon_{r}^{\prime }\right)}^{2}\\ & +1.42\varepsilon^{\prime }\cdot Q_{\mathrm{MUT}}^{-1}-195.12Q_{\mathrm{MUT}}^{-2} \end{array}$$

Substituting the $Q_{\mathrm{MUT}}^{-1}$ and $\varepsilon_{r}^{\prime }$ into (4) gives tanδ. The fitted correlation $R^{2}$ of the Rational Taylor model was calculated to be 0.9665 for the high-frequency resonant point.

## 4. Experimental Part

A prototype sensing probe is fabricated using printed circuit board (PCB) technology, and several solid materials with different dielectric constant are tested. The following sections describe the details of the experiment.

### 4.1. System Working Principle

**Figure 10:**
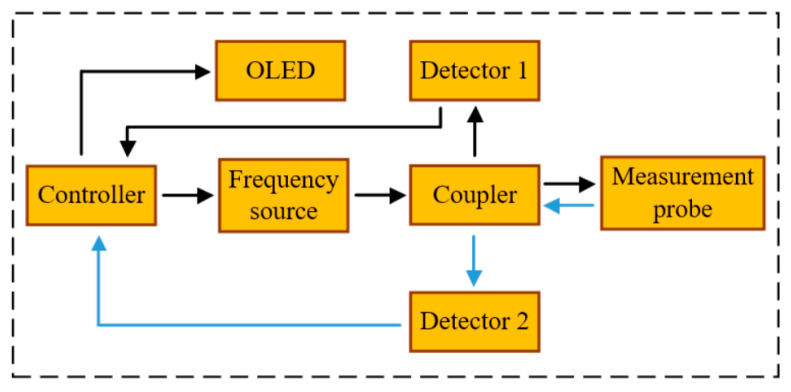
The schematic diagram of the sensor system.

The measurement of resonant frequency points and resonant peak values enables the inversion of the material’s complex dielectric constant, a process which essentially falls within the scope of scalar measurement. In order to circumvent the necessity of utilizing auxiliary measurement apparatus, such as vector network analyzer, a portable and cost-effective independent sensor system was engineered, employing discrete circuit modules. The experimental tests were conducted in conjunction with the sensing probe. The principle block diagram of the system is shown in Figure 10. The specific workflow is as follows:

Step 1: The controller is responsible for receiving the code from the upper computer and controlling the frequency source to generate a sweep signal within a specified frequency band. The signal is first directed to the coupler.

Step 2: Upon entering the coupler, the signal is divided into two paths. One path serves as the reference signal, which is fed into detector 1, while the other path is designated for the measurement signal, which is initially directed to the sensing probe. The coupler subsequently receives the reflected signal from the sensing probe and transmits it to detector 2.

Step 3: Both the reference signal and the reflected signal undergo demodulation before entering the controller, where AD conversion and signal processing operations are performed.

Step 4: The controller then compares the processed data with the internally stored network reference table. The final test results are subsequently displayed on the OLED.

It should be noted that the curve-fitting values of the dielectric constant and the surface-fitting values of the loss tangent in the third part are stored in the controller in the form of a network data table. This provides a reference for the final processing of data by the controller. The frequency source is incapable of achieving equal power sweep signal output throughout the entire frequency range. This has a particularly significant impact on peak attenuation measurement. Consequently, the reference channel fulfils a calibration function. In particular, the signal power balance of the frequency source input coupler is divided into two signals: the reference signal and the measurement signal. The controller is responsible for detecting the power values of all frequency points of the reference signal. It then identifies the point with the maximum power value, records the power difference with other frequency points, and compensates for the reflection signal. This process is essential for achieving the calibration of power imbalance.

### 4.2. Measurement Result

**Figure 11:**
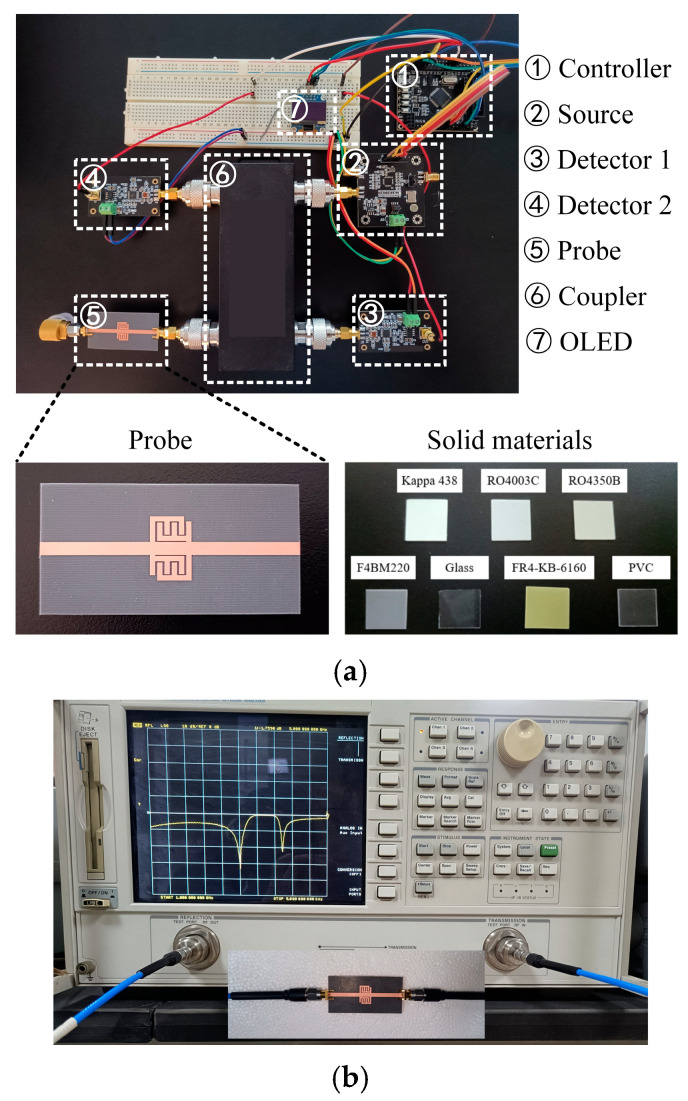
(**a**) Prototype structure of the sensor system. (**b**) VNA-assisted measurement verification.

A prototype of the sensor system is shown in Figure 11a; it can independently complete the measurement of the complex dielectric constant of solid materials. The sensing probe is fabricated on F4BM220 substrate, with $\varepsilon_{r}^{\prime }=2.2$ and tanδ=0.001. The selected connector type is SMA-KE-0.8MM. One end is connected to the sensing probe using a side-insertion method, while the other end is connected to the broadband coupler through a threaded insertion method. The size of solid materials is 15 mm×15 mm×0.5 mm, which is larger than the sensing area; therefore, it completely covers the surface of the sensing area of the probe. These solid materials are all frequency-stable, indicating that their dielectric constant remains unchanged across varying frequencies. Since the sensor system cannot directly display the entire measurement process of the frequency band, a VNA is used for auxiliary measurement verification. In Figure 11b, the VNA is Agilent 8720ET and is calibrated using the SOLT method. After calibrating the sensor system, the reflection coefficient of the probe is first measured unloaded and compared with the simulation results to verify the validity of the sensors. The experimental results are shown in Figure 6, where a slight shift in the resonant frequency is observed due to a fabrication error.

During the experiment, a variety of solid samples are positioned on the designated sensing area. Table 2 summarizes the measurement results of different materials. The reference values for the complex dielectric constants of various solid materials are obtained from the datasheets supplied by the manufacturers. Columns 4 and 5 of Table 2 present the test results obtained from the circuit system, while columns 6 and 7 display the test results from the vector network analyzer. The measurement results indicate that when various solid samples are analyzed, the measurement results obtained from the sensing probe closely align with the reference values, thereby demonstrating high measurement accuracy. Furthermore, the measurement results from the circuit system are consistent with those from the vector network analyzer, validating that the independent circuit system developed in this paper not only offers a cost-effective solution but also fulfills the necessary measurement requirements.

Table 3 shows the comparison between the sensor system proposed in this paper and other similar structures. The dimensions of the sensing probe are normalized to the guided wavelength.

(7) $$size=\frac{A}{\lambda_{g}^{2}}$$

(8) $$\lambda_{g}=\frac{c}{f_{0}\sqrt{\varepsilon_{r}^{\prime }}}$$

where A is the area of the sensing probe, c is the speed of light in vacuum, and $f_{0}$ is the resonant frequency.

In comparison with the dual-frequency structures presented in references [18,33], the sensing probe described in this paper exhibits a reduced normalized size at both resonant frequency points. The normalized size at the low-frequency resonant points is smaller than that of the other references in the table, with the exception of reference [19]. Similarly, the normalized size at the high-frequency resonant point is smaller than those of references [2,27] listed in the table. Overall, the proposed probe demonstrates competitive performance relative to other sensors in terms of the required test samples. The sensing probe, in conjunction with the circuit module, forms a complete sensor system. This sensor system not only offers the advantage of low cost but also facilitates independent and portable measurements.

**Table 3:** Comparison of our work with different techniques in the literature.

Ref.	Resonant Point (GHz)	Probe Size	Material	VNA
[2]	4.48	$4.56\times 10^{-2}$	Liquid	Yes
[15]	2.46	$2.34\times 10^{-2}$	Solid/Liquid	Yes
[18]	2.45/2.86	$6.35\times 10^{-2}$ $9.28\times 10^{-2}$	Liquid	Yes
[19]	1.91	$5.9\times 10^{-3}$	Liquid	Yes
[27]	2.97	$2.3\times 10^{-1}$	solid	Yes
[33]	1.6/2.42	$5.16\times 10^{-2}$ $1.18\times 10^{-1}$	Solid	Yes
Proposed	3/3.92	$2.15\times 10^{-2}$ $3.68\times 10^{-2}$	Solid	No

## 5. Conclusions

A dual-band planar microwave sensor system is proposed for solid complex dielectric constant measurement. The microstrip line and two pairs of E-interdigital structures are used as the host and sensing area, respectively. Two pairs of E-interdigital structures are symmetrically arranged on either side of the microstrip line to reach extreme coupling. It can simultaneously generate two resonant frequency points in one sensing area. Consequently, any environmental effects exhibit equivalent variation at both resonant frequency points, thereby substantiating the efficacy of the proposed sensor system for differential operation, which has the capacity to mitigate the impact of environmental conditions. The proposed sensor system not only has high accuracy, but can also independently measure the complex dielectric constant of solid materials without any auxiliary equipment, significantly reducing the system cost. It has great potential in outdoor portable rapid detection scenarios.

## Figures and Tables

**Table 2:** The measurement results for different materials.

Material	Reference Value		System Measurement		VNA Measurement	
	$\varepsilon_{r}^{\prime }$	tanδ	$\varepsilon_{r}^{\prime }$	tanδ	$\varepsilon_{r}^{\prime }$	tanδ
F4BM220	2.2	0.001	2.17	0.0017	2.18	0.0016
PVC	3.25	0.04	3.21	0.0383	3.21	0.0394
RO4003C	3.55	0.0027	3.48	0.0022	3.50	0.0021
R04350B	3.66	0.004	3.62	0.0037	3.66	0.0035
Kappa438	4.38	0.005	4.33	0.0042	4.35	0.0047
FR4	4.4	0.02	4.36	0.0198	4.41	0.0191

## Data Availability

Data are contained within the article.
